# The impact of endometriosis on work ability in young Australian women

**DOI:** 10.1111/ajo.13683

**Published:** 2023-04-26

**Authors:** Robin J. Bell, Penelope J. Robinson, Marina A. Skiba, Rakibul M. Islam, Chandima Hemachandra, Susan R. Davis

**Affiliations:** ^1^ Women's Health Research Program, School of Public Health and Preventive Medicine Monash University Level 1, 553 St Kilda Road Melbourne 3004 Victoria Australia

**Keywords:** endometriosis, dysmenorrhoea, pelvic ultrasound, work ability

## Abstract

**Background:**

Whereas symptomatic endometriosis may affect work performance, the impact of endometriosis in the general community is not known.

**Aims:**

The associations between endometriosis and each of sick leave and work ability, were investigated in a large sample of non‐healthcare seeking women.

**Materials and Methods:**

This community‐based, cross‐sectional study recruited 6986 women, aged 18–39 years, from three eastern states of Australia between 11 November 2016 and 21 July 2017. Women were identified as having endometriosis if they had undergone a pelvic ultrasound and reported a diagnosis of endometriosis. Working women completed the Work Ability Index.

**Results:**

Participants were predominantly of European ancestry (73.1%) and 46.8% were overweight or had obesity. The prevalence of endometriosis was 5.4% (95%CI 4.9–6.0%) with the highest prevalence of 7.7% (95%CI 6.5 to 9.1%) for women aged 35–39 years. Among the 4618 working women, those with endometriosis had significantly more sick days from work (33.6% reported ≥10 days vs 13.5%, overall χ^2^ *P* < 0.001). Endometriosis was associated with a greater likelihood of poor to moderate work ability, after adjusting for age, body mass index, ethnicity, relationship status, student status, insecure housing, being a carer for another person, parity, ever use of assisted reproductive technologies, and depressed mood (odds ratio 1.90, 95%CI 1.40–2.58, *P* < 0.001).

**Conclusions:**

This study provides new evidence that the negative impact of endometriosis on work attendance and work ability is not limited to women with prevalent symptoms and severe disease, but appears to encompass women across a broader spectrum of this condition in the community.

## INTRODUCTION

Endometriosis is a complex condition with diverse presentations and a broad spectrum of severity. It is characterised by the growth of endometrial tissue outside of the uterine cavity^1^ with classic symptoms including severe pain with menstruation (dysmenorrhoea), chronic pelvic pain, and infertility.^2^ Prevalence estimates for endometriosis vary due to differences in case identification, selection bias and use of point vs cumulative prevalence. Consequently, establishing both the prevalence and impact of endometriosis on women is challenging. Whereas studies that have used diagnostic classification codes for endometriosis‐related hospital admissions have reported endometriosis prevalence estimates between 0.28% and 1.1%,^3^, ^4^, ^5^, ^6^ estimates in the order of 2–7% have been reported for women with an ‘ever diagnosis’ of this condition.^6^, ^7^, ^8^, ^9^

The approach used for case identification influences the determination of the impact of endometriosis on women's lives. Severe disease, necessitating a surgical procedure, has been associated with diminished work productivity.^10^ However, laparoscopy is only recommended for women with negative imaging, eg gynaecological ultrasound, or either contraindications to, or failure of, empirical therapy.^11^, ^12^ Consequently, a significant number of women with this condition are not identifiable by hospital procedure data. The associations between endometriosis and work ability or sick leave have not been reported for a large, community‐based sample that will include women with a broad spectrum of disease severity.

The Grollo‐Ruzzene (GR) Study comprises a representative sample of 6986 Australian women, aged 18 to 39 years.^13^ Thus, this Study has provided a unique opportunity to estimate the prevalence of self‐reported endometriosis and provide a comprehensive estimate of the impact of endometriosis on work ability and overall sick leave taken in the community.

## MATERIALS AND METHODS

### Study population

The GR Study was a community‐based cross‐sectional survey of women, aged 18 to 39 years, living in the eastern Australia states of Victoria, Queensland and News South Wales. Recruitment was achieved using two large electronic data bases between 11 November 2016 and 21 July 2017, as described in detail elsewhere.^13^ Emails invited women to participate in ‘a study of young women's health’ and linked interested women to the study information and consent form. The final 6986 participants were representative of Australian women of the same age in terms of their age and geographic distribution, occupation, education, country of birth and partnership status.^13^ The study was approved by the Monash University Human Research Ethics Committee (CF16/2322–2016001166 (7703)). All participants provided informed consent. The STROBE (Strengthening the reporting of observational studies in epidemiology) statement has been used to report the study findings. All authors had full access to all of the data (including statistical reports and tables) in the study.

### Study questionnaire

The online survey collected a broad range of information including age, height, weight, location of residence, country of birth, ethnicity, relationship status, education, pregnancy and use of assisted reproduction. For most of the questionnaire, participants could not progress without providing a response resulting in an almost complete data set. All participants were required to respond to the question ‘Have you ever had a pelvic ultrasound?’ Participants who responded ‘yes’, were then asked if they had ever been diagnosed with endometriosis. Those who answered ‘yes’ were included in the endometriosis group and triaged to a series of questions pertaining to their diagnosis of endometriosis. These questions aligned with the clinical pathway for the diagnosis of endometriosis in primary care in Australia.^12^ Depressive symptoms were determined by the Beck Depression Inventory‐II^14^ and the findings were dichotomised into moderate–severe vs none‐mild using a cut‐off of ≥20.

### Assessment of work ability and sick leave

Participants were asked if they were working outside the home full‐time or part‐time, with part‐time work defined as less than 38 hours per week. Women were asked how many days they had taken as sick leave from work in the preceding year with options being none, nine days or less, 10–24 days, 25–99 days, or 100–365 days. Work ability was assessed by the Work Ability Index (WAI), a self‐reported instrument developed by the Finnish Institute of Occupational Health. The WAI has seven domains including current work ability in relation to the lifetime best work ability (score 0–10), current work ability in relation to physical demands, mental demands or both (2–10), the number of diseases currently experienced and diagnosed by a physician (1–7), an estimate of the degree of work impairment attributable to disease (1‑6), the extent of absence from work in the previous 12 months due to illness (1‑5), an estimate of work ability in two years (scored as 1, 4 or 7 points) and overall mental resources (1–4). The scores from each domain are summed to give a total score with a higher score indicating better work ability. A score of 7–36 is considered poor to moderate and 37–49 points is considered good to excellent.^15^

### Statistical analysis

Participant characteristics are reported using frequencies and percentages. Between‐group comparisons were made using univariate logistic regression with the outcome variable being endometriosis yes/no. The comparator group for women reporting a diagnosis of endometriosis included those who reported never having had a pelvic ultrasound and those who reported a prior pelvic ultrasound but no endometriosis diagnosis. The relationship between endometriosis and sick leave is reported using frequencies, percentages and χ^2^ test.

The likelihood of having poor‐moderate work ability, compared with good‐excellent work ability was assessed using logistic regression analysis. Model 1 examined an unadjusted association between endometriosis and work ability. Model 2 was adjusted for age, body mass index (BMI), ethnicity, relationship status, student status, insecure housing and being a carer for another person. Model 3 included the characteristics from Model 2 as well as the reproductive factors parity and the use of assisted reproductive technologies. Model 4 included the variables in Model 3 as well as depressive symptoms. Analyses were performed using IBM SPSS Statistics for Windows, version 26 (IBM Corp., Armonk, NY, USA) and Stata 16.0 (Stata Corporation, College Station, TX, USA).

## RESULTS

### Participant characteristics

**Figure 1:**
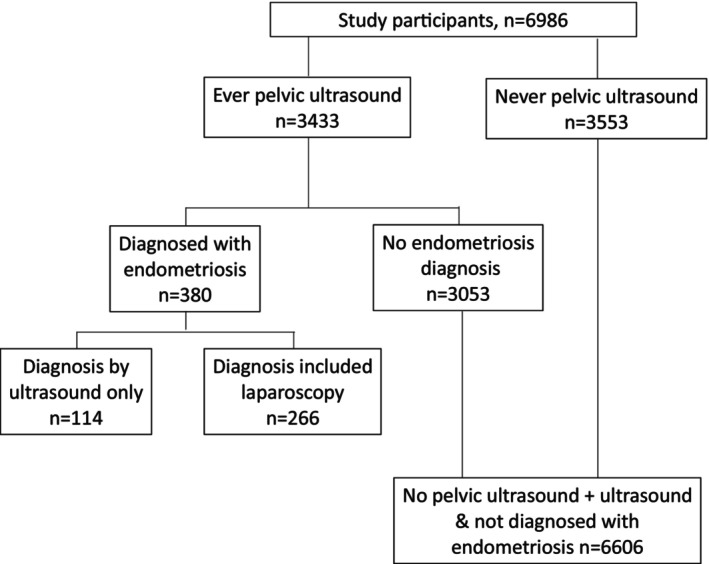
Process by which participants with endometriosis were identified from the 6986 women in the study.

The 6986 participants were predominantly of European ancestry (73.1%), 46.8% were overweight or had obesity, 62.0% were non‐parous (Table 1) and 3433 (49.1%) reported ever having had a pelvic ultrasound (Figure. 1). The prevalence of endometriosis was 5.4% (95%CI 4.9–6.0%).

**Table 1:** Characteristics of study participants at the time of completion of the questionnaire

Factor	No endometriosis *n* = 6606	Yes endometriosis *n* = 380	Total *n* = 6986	OR (from logistic regression)	95% CI OR (from logistic regression)	*P*‐value* (from logistic regression)
	*n* (%)	*n* (%)	*n* (%)			
Age, years						
18 – <25	1997 (30.2)	65 (17.1)	2062 (29.5)	Reference	Reference	Reference
25 – <30	1514 (22.9)	81 (21.3)	1595 (22.8)	1.64	1.18–2.29	0.003
30 – <35	1587 (24.0)	108 (28.4)	1695 (24.3)	2.09	1.53–2.86	<0.001
35 – <40	1508 (22.8)	126 (33.2)	1634 (23.4)	2.57	1.89–3.49	<0.001
Body mass index, kg/m^2^; 36 missing						
Normal 18.5‐ < 25	3126 (47.6)	144 (37.9)	3270 (47.1)	Reference	Reference	Reference
Underweight <18.5	413 (6.3)	16 (4.2)	429 (6.2)	0.84	0.50–1.42	0.52
Overweight 25‐ < 30	1471 (22.4)	92 (24.2)	1563 (22.5)	1.36	1.04–1.78	0.026
Obese ≥30	1560 (23.7)	128 (33.7)	1688 (24.3)	1.78	1.39–2.28	<0.001
Location of residence						
Metropolitan	4434 (67.1)	218 (57.4)	4652 (66.6)	Reference	Reference	Reference
Regional/rural	2172 (32.9)	162 (42.6)	2334 (33.4)	1.52	1.23–1.87	<0.001
Ethnicity						
European ancestry/White	4780 (72.4)	329 (86.6)	5109 (73.1)	Reference	Reference	Reference
Aboriginal/Torres Strait Islander	120 (1.8)	7 (1.8)	127 (1.8)	0.85	0.39–1.83	0.67
Asian	1055 (16.0)	19 (5.0)	1074 (15.4)	0.26	0.16–0.42	<0.001
Polynesian/Melanesian/Māori	81 (1.2)	5 (1.3)	86 (1.2)	0.90	0.36–2.29	0.82
North African/Middle Eastern	91 (1.4)	3 (0.8)	94 (1.3)	0.48	0.15–1.52	0.21
Other/mixed	479 (7.3)	17 (4.5)	496 (7.1)	0.52	0.31–0.85	0.009
Relationship status; 14 missing						
Boy/girl‐friend/single	3260 (49.5)	151 (39.7)	3411 (48.9)	Reference	Reference	Reference
Married/de facto	3332 (50.5)	229 (60.3)	3561 (51.1)	1.48	1.20–1.83	<0.001
Smoking						
Past or never smoker	5673 (85.9)	310 (81.6)	5983 (85.6)	Reference	Reference	Reference
Current smoker	933 (14.1)	70 (18.4)	1003 (14.4)	1.37	1.05–1.80	0.02
Alcohol consumption						
Non‐drinker	2340 (35.4)	146 (38.4)	2486 (35.6)	Reference	Reference	Reference
Consumes alcohol	4266 (64.6)	234 (61.6)	4500 (64.4)	0.88	0.71–1.09	0.24
Working status outside the home						
Not currently working	2108 (31.9)	128 (33.7)	2236 (32.0)	Reference	Reference	Reference
Working full or part‐time	4498 (68.1)	252 (66.3)	4750 (68.0)	0.92	0.74–1.15	0.47
Acting as a carer for another person						
Not a carer for another person	5956 (90.2)	332 (87.4)	6288 (90.0)	Reference	Reference	Reference
Carer for another person	650 (9.8)	48 (12.6)	698 (10.0)	1.32	0.97–1.81	0.08
Student status						
Not a student	4143 (62.7)	263 (69.2)	4406 (63.1)	Reference	Reference	Reference
Full‐ or part‐time student	2463 (37.3)	117 (30.8)	2580 (36.9)	0.75	0.60–0.94	0.01
Housing financial security; 35 missing						
Secure	5459 (83.1)	313 (82.4)	5772 (83.0)	Reference	Reference	Reference
Insecure	1112 (16.9)	67 (17.6)	1179 (17.0)	1.05	0.80–1.38	0.72
Depressive symptoms#						
None/mild	4674 (70.8)	233 (61.3)	4907 (70.2)	Reference	Reference	Reference
Moderate/severe (BDI > =20)	1932 (29.2)	147 (38.7)	2079 (29.8)	1.53	1.23–1.89	<0.001
Ever used assisted reproduction						
No	6288 (95.2)	318 (83.7)	6606 (94.6)	Reference	Reference	Reference
Yes	318 (4.8)	62 (16.3)	380 (5.4)	3.86	2.87–5.18	<0.001
Parous						
Non‐parous	4127 (62.5)	206 (54.2)	4333 (62.0)	Reference	Reference	Reference
Has at least one child	2479 (37.5)	174 (45.8)	2653 (38.0)	1.41	1.41–1.73	0.001
Currently pregnant						
No	6319 (95.7)	352 (92.6)	6671 (95.5)	Reference	Reference	Reference
Yes	287 (4.3)	28 (7.4)	315 (4.5)	1.75	1.17–2.62	0.006

BDI, Beck Depression Index; OR, odds ratio.

* *P*‐value from univariate logistic regression with the outcome variable endometriosis yes/no.

### Characteristics of women reporting endometriosis

**Figure 2:**
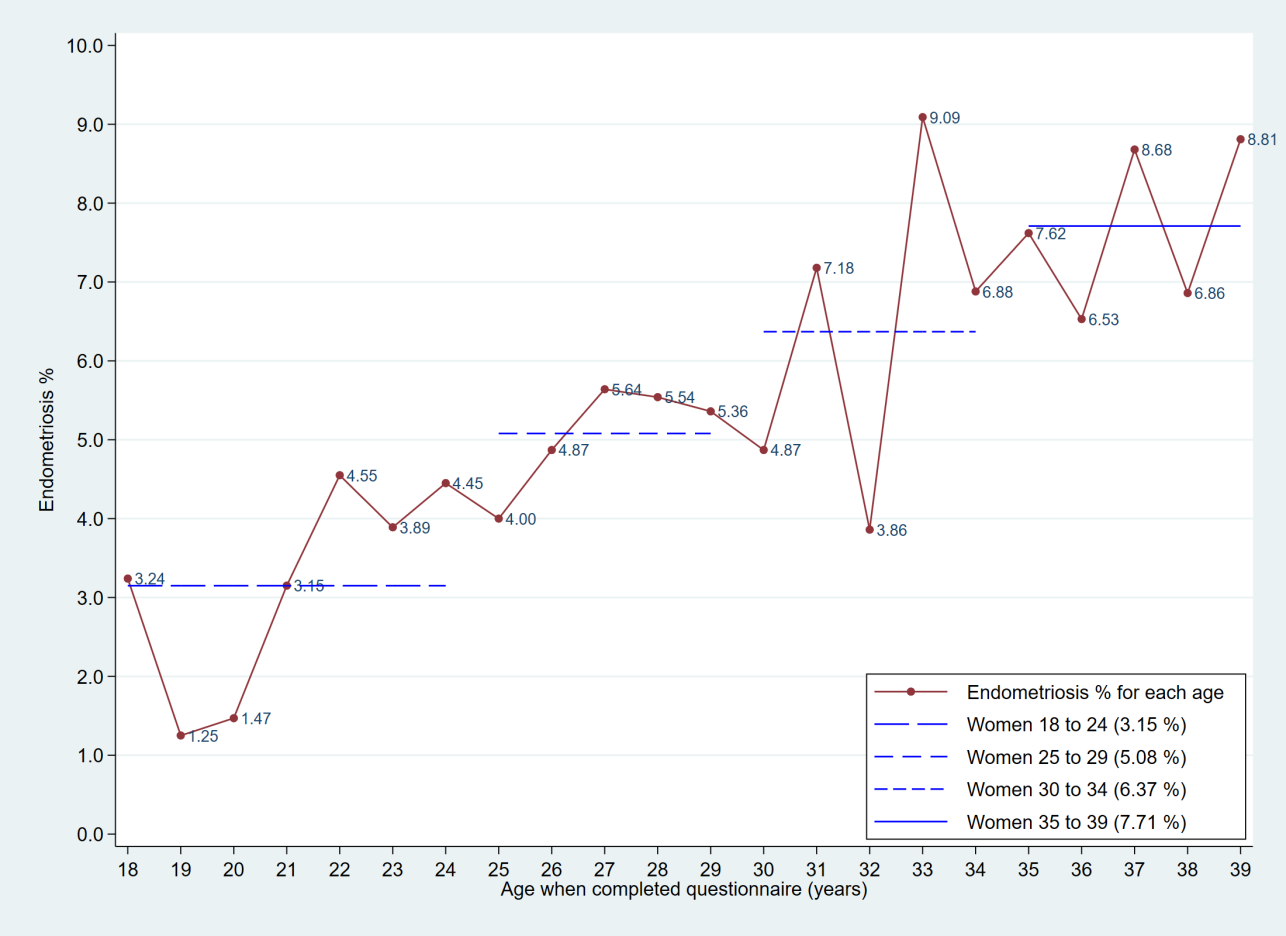
The percentage of women who reported a diagnosis of endometriosis by their age when they completed the study questionnaire.

Of the 380 women reporting endometriosis, 70% said their diagnosis included a laparoscopy. The age‐specific prevalence increased with increasing age at the time women completed the questionnaire, from 3.2% (95%CI 2.5 to 4.0%) for women aged 18 to <25 years to 7.7% (95%CI 6.5 to 9.1%) for women aged 35–<40 years (Figure. 2). Two‐thirds of the 380 women with endometriosis reported being diagnosed before the age of 25 years.

Women who reported endometriosis were older (mean age 30.92 (SD 5.8) years vs 28.67 (SD 6.3), *P* < 0.001) and had a higher BMI (median 26.1 kg/m^2^ vs 24.5 kg/m^2^, *P* < 0.001). They were also more likely to be partnered (*P* < 0.001), parous (*P* = 0.001), currently pregnant (*P* = 0.006), have used assisted reproduction (*P* < 0.001), live in a rural than metropolitan setting (*P* < 0.001), have moderate–severe depressive symptoms (*P* < 0.001), and smoke (*P* = 0.02). They were less likely to identify as being of Asian ethnicity (*P* < 0.001).

### Endometriosis, sick leave and work ability

Of the 4750 women working outside the home, 4618 (97%) provided information about the hours worked and were included in the analysis pertaining to work ability (Table 2). A greater proportion of women without endometriosis reported no sick leave in the past year compared with those reporting endometriosis (47.0% vs 28.7%), while 10 or more days of sick leave was reported by 13.5% and 33.6%, respectively (overall χ^2^ *P* < 0.001).

**Table 2:** Absence from work in the previous 12 months due to illness and self‐reported work ability

	No endometriosis *n* = 4371 (94.7%)	Endometriosis *n* = 247 (5.3%)	Total *N* = 4618
Number of days of sick leave in the last year*	*n* (%)	*n* (%)	*n* (%)
100–365	46 (1.1)	6 (2.4)	52 (1.1)
25–99	131 (3.0)	23 (9.3)	154 (3.3)
10–24	412 (9.4)	54 (21.9)	466 (10.1)
Nine days or less	1726 (39.5)	93(37.7)	1819 (39.4)
None	2056 (47.0)	71 (28.7)	2127 (46.1)
Work ability *n* (%)			
Good‐excellent	3248 (74.3)	145 (58.7)	3393 (73.5)
Poor‐moderate	1123 (25.7)	102 (41.3)	1225 (26.6)

Percentages reported by column.

* Statistically significant χ^2^ test *P* < 0.001.

Endometriosis was associated with a significantly greater likelihood of WAI score of poor to moderate work ability (odds ratio (OR) 2.03, 95%CI 1.57–2.64, *P* < 0.001). This association did not meaningfully change after adjustment for age, BMI, ethnicity, relationship status, student status, insecure housing, being a carer for another person, parity, ever use of assisted reproductive technologies, and depressed mood (OR 1.90, 95%CI 1.40–2.58, *P* < 0.001).

## DISCUSSION

Our study of 4618 young women working outside the home found endometriosis to be associated with greater absence from work due to illness and an independent risk factor for lower self‐assessed work ability, even when multiple other factors that influence work ability were taken into account. Although we did not identify the symptoms for which women took sick leave, the effects of endometriosis have been reported to extend to both psychological and physical wellbeing and may manifest as fatigue, physical impairment low sleep quality and reduced quality of life.^16^

To our knowledge, this is the first study to report the impact of endometriosis on work ability in a community‐based sample, with adjustment for an array of parameters, including moderate to severe depression. A similar association between endometriosis and poor work ability was reported in a Danish study of women with self‐reported disease.^17^ However, the findings are limited by potential selection bias, as participants were recruited to a study of pain and work ability.^17^ Hence, it is not surprising that the authors reported more frequent absences from work due to illness in both controls and women with endometriosis than in our study.^17^ A multi‐national study of women scheduled for laparoscopy reported endometriosis disease severity and pelvic pain to be associated with lower work productivity.^10^ The investigators noted there was a high likelihood of over‐representation of women with more advanced disease in their study as participants were recruited from tertiary referral centres and their response rate was relatively low.^10^

Our study sample was not only large and unselected for the condition of interest, but also representative of the population from which participants were recruited.^13^ The group we considered to have endometriosis were women who had been told they had a diagnosis of endometriosis. This group would have included women who responded to initial medical therapy and did not progress to laparoscopy. Although our approach of not requiring a laparoscopy to be included in our group with endometriosis is consistent with the guidelines of the European Society of Human Reproduction and Embryology,^11^ this guideline has not been endorsed by the Royal Australian and New Zealand College of Obstetricians and Gynaecologists, which currently does require laparoscopic confirmation of the diagnosis. Despite this, our prevalence estimate of endometriosis was in accordance with that reported by the Australian Longitudinal Study of Women's Health^18^ and other prior large, cross‐sectional studies.^7^, ^8^, ^19^ Our inclusive definition provides a ‘real world’ picture of the impact of an ever‐diagnosis of endometriosis on young working women.

It is to be expected that ever being diagnosed with endometriosis might have a cumulative lifetime impact. In our study the majority of women were diagnosed before the age of 25 years, and therefore their disease is likely to have negatively affected their education, social engagement and career‐development at a critical life stage.^20^ Furthermore, as endometriosis often requires multiple surgeries, it commonly affects other organs including the bladder and bowel, is a frequent cause of infertility, and often requires long‐term medication to prevent recurrence; thus, the effects of this condition linger well beyond the time of diagnosis and initial treatment.^20^ However, past studies have primarily focused on symptomatic women and those attending for diagnostic procedures.^10^, ^17^, ^20^, ^21^, ^22^ As expected, there was increased use of assisted reproductive technology in the endometriosis group but also a statistically higher number of children in this group. Despite the recognised association between endometriosis and reduced fertility,^23^ that the women with endometriosis in our study were more likely to be parous than those who did not report a diagnosis of endometriosis, is likely explained by the fact that Australian women have access to assisted reproductive technologies and the women with endometriosis in our study were older than those without endometriosis.

This could also have accounted for the increased use of sick leave, during pregnancy and for parental leave. Thus, our study provides new evidence that the negative impact of endometriosis on work attendance and work ability is not limited to women with prevalent symptoms and severe disease, but encompasses women across the broad spectrum of this condition, including women previously treated, but not currently severely symptomatic.

It was not appropriate for us to explore ‘risk factors’ for endometriosis in our study for several reasons. The participants' characteristics were from the time of completion of the questionnaire, not at the time of diagnosis of endometriosis. Additionally, relationship status, parity, student status and working outside the home, will be confounded by age, as well as with each other, and some of the characteristics are a ‘consequence’ rather than a ‘risk factor’ for endometriosis, such as parity and use of assisted reproductive technology.

Our goal was to identify women ever diagnosed with endometriosis who carried the label of having the condition. This was purposely not a study of chronic pelvic pain as we did not ask about prevalent symptoms. However, we used ‘ever having had an ultrasound’ to filter out spurious diagnoses, as ultrasound is the first line investigation for this condition and readily accessible for Australian women presenting with symptoms.^12^ It is unlikely that we have over‐estimated the prevalence of endometriosis. Rather, it is more likely that the prevalence was under‐estimated, as we will have missed some women whose symptoms responded to empirical treatment, such as the oral contraceptive pill, and were never referred for a pelvic ultrasound.^24^ While we relied on self‐reported diagnosis of endometriosis, this approach has been found to have robust validity.^25^, ^26^ With respect to work ability, we would have missed women with severe symptoms that resulted in their leaving the workforce. As access to diagnostic services for endometriosis differs between countries, our findings should be generalised beyond Australia with caution.

Our analysis of data from this representative sample of Australian women, aged 18 to 39 years, shows that participants who self‐identified as having endometriosis have more days of sick leave and lower work ability than other women of similar age. While the deleterious effect of severe endometriosis on work ability has been previously recognised, it is important to consider the adverse effects across the full disease spectrum. Clinicians should also be mindful of this when caring for women affected by this condition and policy decisions need to be made to promote the health and work ability of young women who make important contributions in the work force.

## Author contributions

SRD, RJB and MAS conceived and designed the study. SRD and MAS collected the data and PJR and RJB conducted the data analysis and prepared the figures. The data interpretation was done by all co‐authors. PJR, SRD, RJB and CDH drafted the manuscript which was reviewed by all co‐authors.

## Funding information

The funders had no role in study design, data collection, data analysis, data interpretation, or writing of the report. All authors had full access to all the data in the study and had final responsibility for the decision to submit for publication.

## Data Availability

The data for the Grollo‐Ruzzene Study is not presently available to be shared due to participant privacy considerations.
